# Changes in food cravings, dietary quality, body composition, and dietary intake during GLP-1 receptor agonist therapy: The CRAVE study

**DOI:** 10.1016/j.obpill.2026.100292

**Published:** 2026-06-29

**Authors:** Demsina Babazadeh, Stephanie Therrien, Angela K. Fitch, Francene M. Steinberg

**Affiliations:** a Graduate Group in Nutritional Biology, University of California, Davis, Davis, CA, USA; b Department of Nutrition, University of California, Davis, Davis, CA, USA; c Knownwell Clinic, Needham, MA, USA

**Keywords:** Diet quality, Food cravings, GLP1, Obesity, Semaglutide, Tirzepatide

## Abstract

**Objective:**

To evaluate changes in diet quality, food cravings, dietary intake, and body composition during 24 weeks of GLP-1 RA OMM therapy in a real-world clinical setting.

**Methods:**

In this prospective observational study, adults with obesity initiating semaglutide or tirzepatide were followed for 24 weeks (analytic n = 28). Participants did not receive structured nutrition intervention. Dietary intake was assessed using food records, and diet quality was evaluated using the Healthy Eating Index (HEI-2020). Food cravings were measured using the Food Cravings Inventory-III questionnaire. Body composition was assessed via bioelectrical impedance analysis.

**Results:**

GLP-1 RA OMM therapy was associated with significant reductions in body weight, adiposity, and BIA-estimated skeletal muscle mass (all P < 0.001); with approximately one-quarter of weight loss attributable to estimated skeletal muscle mass. Total energy intake decreased significantly, with parallel reductions in macronutrient intake. Higher absolute protein intake was associated with greater preservation of estimated skeletal muscle mass (r = 0.41, P = 0.0498). Diet quality did not significantly improve in our sample and food cravings were unchanged overall, although higher baseline cravings were associated with greater reductions over time (r = −0.69, P < 0.001). Significant declines in intake were observed across multiple micronutrients.

**Conclusions:**

In this small prospective cohort, GLP-1 RA OMM therapy in a real-world setting without structured nutrition support, was associated with substantial weight and fat loss accompanied by reductions in estimated skeletal muscle mass, energy intake, and micronutrient consumption, without improvement in diet quality or food cravings. Given the modest sample size and participant attrition, these findings should be considered exploratory and hypothesis-generating. The observed changes in dietary intake and body composition may highlight a critical gap in obesity management that warrants further investigation in larger, adequately powered studies to evaluate the role of nutrition-focused care, particularly strategies targeting protein adequacy and nutrient density during pharmacologic weight loss.

**Clinical trials registration:**

NCT06467604.

## Graphical abstract

**Figure:**
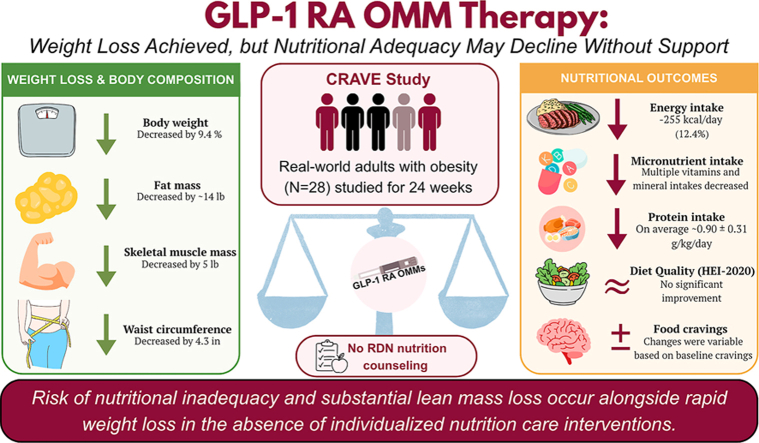

## 1 Introduction

Glucagon-like peptide-1 receptor agonists have transformed the clinical management of obesity, producing substantial and sustained reductions in body weight across randomized trials and real-world settings [1]. For the purposes of this study, glucagon-like peptide-1 (GLP-1) receptor agonists and dual glucose-dependent insulinotropic polypeptide (GIP)/GLP-1 receptor agonists obesity management medications (OMMs) are collectively referred to as GLP-1 RA OMMs. These agents exert their effects through complementary physiologic pathways, including delayed gastric emptying, enhanced satiety signaling, and central regulation of appetite and ingestive behavior via hypothalamic circuits and various brain regions [[2], [3], [4]]. Accordingly, GLP-1 RA OMMs have been rapidly integrated into clinical care, with therapeutic goals extending beyond weight loss to encompass broader cardiometabolic risk reduction [5,6].

Despite these advances, the behavioral and dietary mechanisms underlying weight loss during GLP-1 RA OMM therapy remain incompletely characterized. To date, clinical trials have predominantly focused on changes in body weight and metabolic endpoints, with comparatively limited assessment of food-related behaviors that directly influence energy intake [7,8]. Among these, food cravings defined as intense, specific desires for foods represent a critical yet underexamined construct, given their associations with dietary patterns and overall diet quality, including metrics such as the Healthy Eating Index (HEI) [9]. Measures of dietary intake and quality are increasingly recognized as central to cardiometabolic health yet remain inconsistently assessed in obesity pharmacotherapy research [7]. In parallel, changes in body composition during pharmacologic weight loss, particularly skeletal muscle loss and its implications for resting metabolic rate (RMR), have received greater attention than behavioral and dietary outcomes [[10], [11], [12]]. Together, these gaps underscore the need for more comprehensive phenotyping approaches that extend beyond total weight loss to capture both behavioral and physiologic adaptations to therapy. This limitation is consistent with a growing body of literature emphasizing that dietary intake, food-related behaviors, and overall diet quality are frequently under-assessed in clinical and translational obesity research [8,13,14].

To address these limitations, the present pilot study prospectively evaluated changes in dietary intake, diet quality, and food cravings over 24 weeks among adults with overweight and obesity initiating GLP-1 RA OMM therapy in a real-world clinical setting. By prioritizing these behavioral outcomes alongside physiologic measures, this study aims to elucidate key dietary and behavioral mechanisms and their clinical implications in GLP-1 RA OMM–associated weight loss.

## 2 Methods

### 2.1 Study design

This prospective, observational study evaluated behavioral and physiologic responses to GLP-1 RA OMM therapy in a real-world clinical setting. Adults aged 18–70 years with BMI ≥30 kg/m^2^, or ≥27 kg/m^2^ with at least one weight-related comorbidity, initiating GLP-1 RA OMM therapy were recruited from a specialty obesity clinic and followed for 24 weeks. Participants were determined to be eligible for GLP-1 RA OMM by their provider and recruited by the study team after medical eligibility. Participants were required to complete online dietary and behavioral assessments and commit to in-clinic body composition measurements. Key exclusion criteria included prior bariatric surgery, recent use of GLP-1 RA OMM–based therapies, diabetes, active malignancy, significant psychiatric conditions, or other medical conditions that could interfere with study participation or interpretation of outcomes. A total of 45 participants were enrolled between 2022 and 2025, including 28 with complete data across baseline, midpoint, and 24-week assessments who comprised the primary analytic sample. All participants provided informed consent, and the study protocol was approved by the institutional review board at the University of California Davis.

### 2.2 Sample size considerations

Sample size estimates were based on prior evidence demonstrating a moderate effect size for changes in food cravings (Cohen's d ≈ 0.56) [15]. A total of 116 participants was estimated to provide 85% power at a two-sided α = 0.05, with a target enrollment of 150 accounting for ∼25% attrition. Despite these projections, recruitment yielded a final sample of 28 participants over a two-year period, reflecting the pragmatic challenges of enrolling patients initiating GLP-1 RA OMM therapy in real-world clinical settings.

### 2.3 Clinical care and assessments

Participants were prescribed semaglutide or tirzepatide as part of routine clinical care, with medication selection, dose escalation, and maintenance determined by treating clinicians irrespective of this study. Assessments were conducted at baseline, midpoint (8–12 weeks), and 24 weeks, aligned with standard titration schedules and prior GLP-1 RA OMM trials [2,3]. Participants did not receive registered dietitian nutritionist (RDN) led nutrition intervention as part of the study protocol, to reflect real-world clinical settings in which comprehensive, multidisciplinary obesity care is not consistently implemented. Participants attended routine clinical visits as determined by standard operating procedures of the clinic and additional study data were collected remotely via electronic questionnaires.

### 2.4 Dietary intake and diet quality

Dietary intake was assessed using 3-day food records collected at each time point via the RxFood™ application. Participants recorded all foods and beverages consumed over three consecutive days. Food records were reviewed and transcribed into Nutrition Data System for Research by trained RDNs in collaboration with Brigham and Women's Hospital in Boston, MA. Diet quality was assessed using the HEI, calculated according to established scoring algorithms [16,17]. Due to resource constraints, dietary data processing was conducted for participants completing at least two timepoints, resulting in HEI availability for a subset of the baseline cohort.

### 2.5 Food cravings and behavioral measures

Food cravings were assessed using the Food Cravings Inventory-III (FCI-III), which measures frequency of cravings across food categories, with higher scores indicating greater craving frequency [18].

Additional measures included the MESA Neighborhood Healthy Food Availability Scale [19,20], the 2-item Hunger Vital Sign for food security [21], the Binge Eating Disorder Screener-7 [22], hunger visual analogue scale [23], and the Paffenbarger Physical Activity Questionnaire, commonly been used to assess leisure-time physical activity [24].

### 2.6 Body composition and resting metabolic rate

Body composition was assessed using the Seca mBCA 554 bioelectrical impedance analysis (BIA) system at baseline, midpoint, and 24 weeks. The device provides estimates of body composition compartments, including fat mass and skeletal muscle mass (SMM), derived from prediction algorithms based on impedance measures [25]. Height and weight were measured using standardized procedures. Resting metabolic rate (RMR) was estimated by the device using proprietary prediction equations and was not a direct measure of energy expenditure consistent with prior work evaluating BIA-based RMR estimates against indirect calorimetry in some populations [26]; accordingly, it was evaluated as an exploratory outcome.

### 2.7 Statistical analysis

Age was calculated using date of birth and baseline date or estimated using the enrollment period when unavailable. Continuous variables are presented as mean (SD) and categorical variables as n (%). Between-group differences were assessed using independent t-tests or chi-square/Fisher's exact tests, while within-participant changes were evaluated using paired t-tests and between-group differences in change using independent t-tests. Associations were assessed using Pearson correlations with 95% confidence intervals. Dietary intake was analyzed as both absolute (g/day) and body weight–adjusted (g/kg). Analyses were conducted using complete-case data. P values are presented without adjustment for multiple comparisons and should be interpreted as descriptive, particularly for secondary, subgroup, and individual-item analyses. Statistical significance was defined as two-sided P < 0.05.

## 3 Results

### 3.1 Participant baseline characteristics

**Fig. 1:**
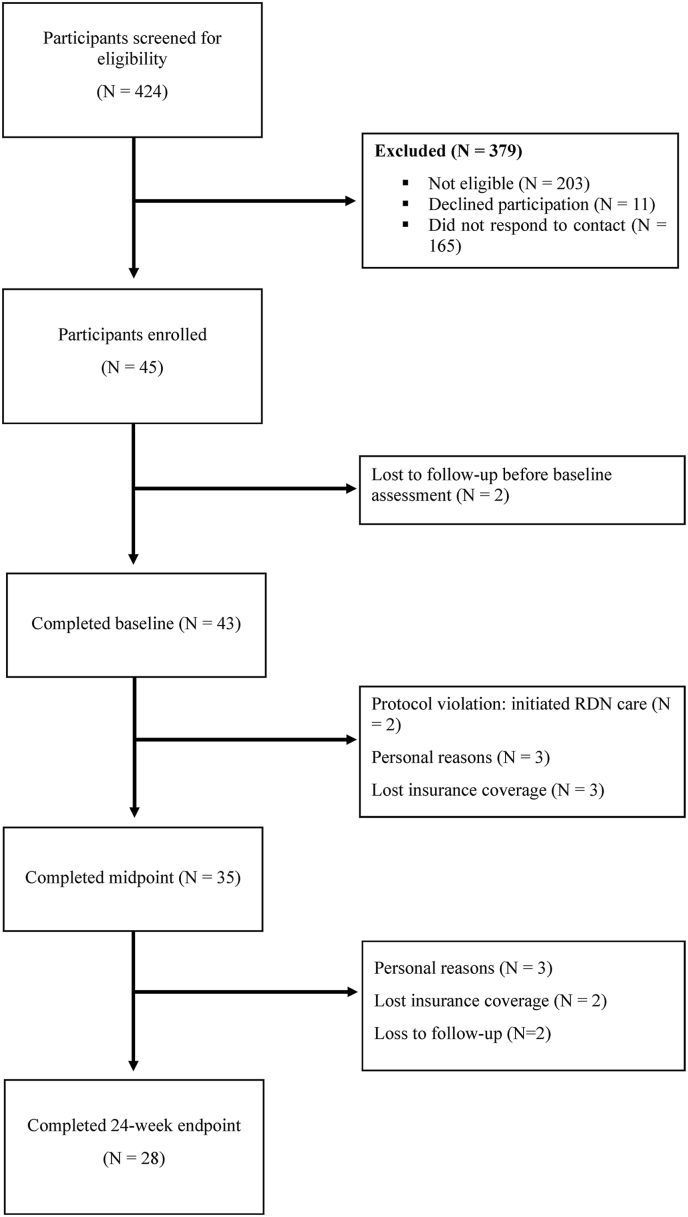
**Participant flow diagram for the CRAVE prospective study.** Participants were screened for eligibility, enrolled, and followed through baseline, midpoint (8–12 weeks), and 24-week endpoint assessments. Attrition at each stage is shown with reasons for withdrawal, including protocol violations (initiation of registered dietitian care), personal reasons, and loss of insurance coverage.

A total of 43 participants were enrolled at baseline; 35 completed the midpoint visit and 28 completed the 24-week endpoint (Fig. 1). Baseline characteristics were assessed in the full cohort (n = 43), including 18 participants initiating semaglutide and 25 initiating tirzepatide (Table 1). Groups were generally comparable at baseline, although fat mass percentage was higher among semaglutide-treated participants (49.0% vs 42.8%, P = 0.013).

**Table 1:** Baseline characteristics of participants in the CRAVE study by GLP-1RA OMM type.

Characteristic	Overall *N=43*	Semaglutide *N=18*	Tirzepatide *N=25*	P value
**Age, years**	43.0 (11.8)	42.5 (13.7)	43.4 (10.6)	>0.9
**Sex**				0.064
Female	38 (88.4%)	18 (100.0%)	20 (80.0%)	
Male	5 (11.6%)	0 (0.0%)	5 (20.0%)	
**Weight, lb**	210.3 (43.0)	217.0 (45.0)	205.7 (42.0)	0.4
Missing	3	2	1	
**BMI, kg/m^2^**	34.7 (6.5)	36.2 (6.9)	33.6 (6.2)	0.3
Missing	3	2	1	
**Waist circumference, in**	41.4 (5.1)	41.6 (5.0)	41.2 (5.2)	0.6
Missing	3	2	1	
**Fat mass, %**	45.3 (7.7)	49.0 (6.6)	42.8 (7.5)	0.013
Missing	3	2	1	
**Fat mass, lb**	97.3 (33.2)	108.3 (36.6)	89.9 (29.2)	0.11
Missing	3	2	1	
**Estimated SMM lb**	53.0 (9.6)	50.7 (6.1)	54.4 (11.2)	0.4
Missing	3	2	1	
**BIA-estimated RMR, kcal/day**	1677.6 (240.3)	1669.1 (228.8)	1683.2 (252.4)	>0.9
Missing	3	2	1	
**HEI-2020 score**	59.2 (10.4)	59.7 (11.0)	58.9 (10.3)	>0.9
Missing	11	7	4	
**Hunger (VAS)**	36.0 (21.9)	32.9 (25.7)	38.2 (19.1)	0.4
**Fullness (VAS)**	51.7 (27.1)	50.9 (28.2)	52.3 (26.8)	0.8
**Satisfaction (VAS)**	54.4 (21.8)	56.0 (22.8)	53.2 (21.5)	>0.9
**Desire to eat (VAS)**	51.0 (24.7)	46.0 (24.5)	54.7 (24.6)	0.2

Data are presented as mean (SD) for continuous variables and n (%) for categorical variables. Sample sizes vary by variable due to missing data. Dietary intake data used to calculate Healthy Eating Index (HEI-2020) scores were available for a subset of participants who completed 3-day food records at baseline. Between-group comparisons were performed using Wilcoxon rank-sum tests for continuous variables and Fisher's exact tests for categorical variables. BMI, body mass index; HEI, healthy eating index; RMR, resting metabolic rate; SMM, skeletal muscle mass; VAS, visual analogue scale.

Semaglutide (Wegovy) and tirzepatide (Zepbound).

HEI-2020 data were available for 32 participants with sufficient dietary records across at least two study time points. Participants with available data had significantly lower baseline BMI than those without dietary data (33.7 vs 37.4 kg/m^2^, P = 0.034), indicating potential selection bias in complete-case dietary analyses (Supplemental Table 1). Of the 43 participants enrolled, 35 (81.4%) completed the midpoint assessment and 28 (65.1%) completed the 24-week endpoint assessment, resulting in an overall attrition rate of 34.9%. Non-completion was due to protocol violation (n = 2), personal reasons (n = 6), loss of insurance coverage (n = 5), or loss to follow-up (n = 2). Participants who completed the endpoint (n = 28) had significantly lower baseline BMI than non-completers (n = 15) (33.6 vs 36.6 kg/m^2^, P = 0.038) (Supplemental Table 2). Other baseline characteristics, including age, sex, and medication type, were comparable by completion status. Analytic sample sizes were n = 28 for paired diet quality and food craving analyses, n = 23 for body composition and protein analyses, and n = 14 for physical activity analyses.

### 3.2 Food cravings

**Fig. 2:**
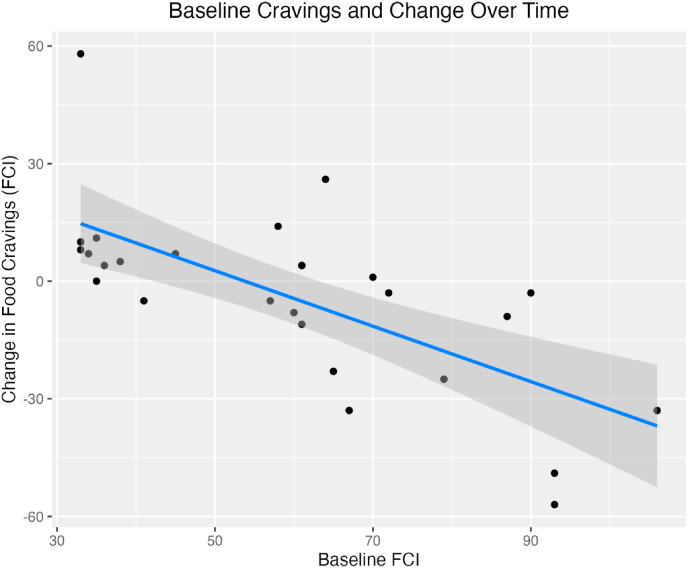
Each point represents an individual participant (N = 28). The solid line represents the fitted linear regression, with shaded area indicating the 95% confidence interval. Change in food cravings reflects within-subject difference in Food Cravings Inventory (FCI) score from baseline to 24 weeks, with negative values indicating reductions in cravings.

Total FCI-III scores did not significantly change from baseline to endpoint (58.6–55.2; Δ = −3.39, P = 0.727). Despite the absence of a group-level effect, substantial inter-individual variability was observed. Baseline FCI-III scores were strongly inversely associated with change in FCI-III (r = −0.69, P < 0.001), indicating that participants with higher baseline cravings experienced greater reductions over time (Fig. 2). Exploratory analyses of individual FCI-III food items demonstrated substantial inter-individual variability in craving responses over 24 weeks (Supplemental Fig. 1). Most item-specific cravings did not significantly change from baseline to endpoint (Supplemental Table 3). Among individual items, brownie cravings showed a nominal reduction from baseline to endpoint (2.321 ± 1.442 vs. 1.607 ± 0.956; mean change, −0.714 ± 1.675; P = 0.043).

### 3.3 Diet quality and intake

**Fig. 3:**
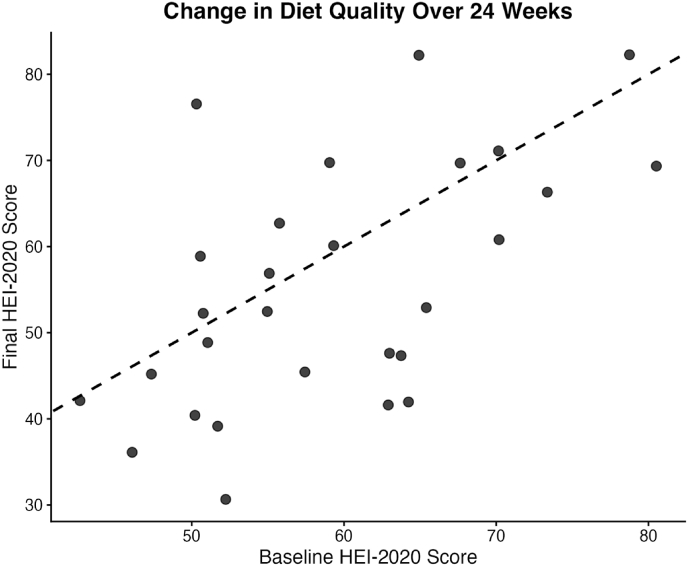
Each point represents an individual participant (N = 28). The dashed line represents the line of identity (y = x), indicating no change between baseline and 24 weeks. Values above the line reflect improvements in HEI-2020 score, whereas values below the line indicate declines in diet quality over time.

Overall diet quality, assessed using HEI-2020, declined modestly over the intervention period but did not reach statistical significance (59.3–55.4; Δ = −3.88, P = 0.070) (Fig. 3). When stratified by medication, participants receiving semaglutide (n = 9) demonstrated no significant change in HEI (Δ = −1.19, P = 0.813), whereas those receiving tirzepatide (n = 19) experienced a significant decline (Δ = −5.16, P = 0.042). However, the between-group difference in change was not statistically significant (P = 0.468) (Supplemental Fig. 2). The prevalence of food insecurity in this cohort was low (n = 3), precluding meaningful statistical comparisons. Baseline diet quality was not associated with neighborhood food access as measured by the MESA score (r = 0.16, P = 0.43).

Total energy intake decreased significantly from baseline to endpoint (−255.26 ± 490.17 kcal/day, P = 0.008), accompanied by reductions in carbohydrate (−24.61 ± 58.72 g/day, P = 0.014) and fat intake (−11.26 ± 25.55 g/day, P = 0.037). Absolute protein intake also declined (−8.73 ± 26.44 g/day), though this did not reach statistical significance (P = 0.094). Average protein intake normalized to body weight was 0.90 ± 0.31 g/kg/day. Despite reductions in absolute energy and macronutrient intake, macronutrient composition expressed as percent energy intake remained stable from baseline to endpoint. Percent energy derived from carbohydrate (37.7 ± 6.3% vs 37.8 ± 6.2%, P = 0.847), protein (23.4 ± 5.9% vs 24.7 ± 5.1%, P = 0.531), and fat (37.2 ± 5.4% vs 37.0 ± 5.7%, P = 0.991) did not significantly change over the 24-week intervention period (Supplemental Table 4).

Significant reductions in micronutrient intake were observed across several vitamins and minerals, including magnesium (−47.35 ± 83.19 mg, P = 0.005), potassium (−380.49 ± 687.85 mg, P = 0.007), iron (−1.92 ± 5.28 mg, P = 0.044), zinc (−1.23 ± 2.99 mg, P = 0.049), copper (−0.22 ± 0.35 mg, P = 0.003), selenium (−12.91 ± 31.78 mcg, P = 0.046), and B vitamins such as niacin (−3.98 ± 6.52 mg, P = 0.006), vitamin B6 (−0.43 ± 0.80 mg, P = 0.002), pantothenic acid (−0.90 ± 1.36 mg, P = 0.002), and folate (−50.01 ± 123.89 mcg, P = 0.035) (Supplemental Table 4).

### 3.4 Body composition and anthropometrics

Significant reductions in body weight and adiposity were observed over 24 weeks (Supplemental Table 5). Among participants with paired body composition data (n = 23), mean total body weight loss was 9.4% ± 5.0% over 24 weeks. Overall, 82.6% of participants achieved ≥5% weight loss, 47.8% achieved ≥10% weight loss, 17.4% achieved ≥15% weight loss, and 4.3% achieved ≥20% weight loss in 24 weeks. Among participants with paired data (n = 23), body weight decreased by 19.0 ± 11.9 lb (P < 0.001), corresponding to a reduction in BMI of 3.1 ± 2.0 kg/m^2^ (P < 0.001). Waist circumference decreased by 4.3 ± 2.3 inches (P < 0.001). Fat mass, as measured by BIA, decreased by 14.4 ± 10.1 lb and 3.4 ± 2.6 percentage points (both P < 0.001). Concurrently, estimated SMM decreased by 5.0 ± 5.3 lb and BIA-estimated RMR declined by 88.2 ± 51.8 kcal/day (both P < 0.001). However, estimated SMM as a proportion of total body weight was relatively preserved over time (25.6% ± 4.1% at baseline versus 26.6% ± 4.3% at 24 weeks). Among total tissue changes, approximately one-quarter of weight loss was attributable to reductions in estimated SMM (27.4% ± 15.7%). Exploratory analyses stratified by medication type demonstrated similar reductions in body weight, estimated SMM, and fat mass among participants receiving semaglutide (Wegovy) and tirzepatide (Zepbound) (Supplemental Table 6). Although significant within-group reductions were observed for both medications, no statistically significant differences in body composition or weight change outcomes were detected between medication groups (all P ≥ 0.37).

### 3.5 Protein intake and skeletal muscle mass

**Fig. 4:**
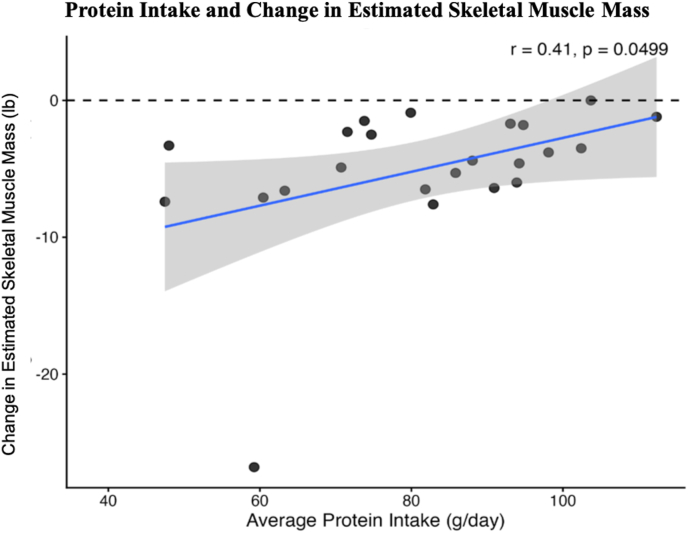
Each point represents an individual participant (N = 28). The solid line represents the fitted linear regression, with shaded area indicating the 95% confidence interval. Change in estimated skeletal muscle mass from BIA reflects within-subject difference from baseline to 24 weeks. Pearson correlation coefficient (r) and corresponding two-sided P value are shown.

Baseline protein intake normalized to body weight (g/kg/day) was not significantly associated with changes in the estimated SMM (r = 0.28, 95% CI −0.15 to 0.62; P = 0.199). Similarly, average protein intake normalized to body weight across study timepoints was not associated with change in estimated SMM (r = 0.21, 95% CI −0.22 to 0.57; P = 0.341). In contrast, higher absolute protein intake (g/day) was linked to greater preservation of estimated SMM (r = 0.41, 95% CI 0.002 to 0.71; P = 0.0498) (Fig. 4), although significance was attenuated in sensitivity analyses excluding an influential observation (r = 0.40, P = 0.066). Physical activity was not related to estimated SMM change (r = −0.18, P = 0.533). GLP-1 RA OMM use over 24 weeks was associated with substantial reductions in body weight and adiposity, accompanied by parallel declines in estimated SMM, without improvement in diet quality or overall food cravings.

## 4 Discussion

In this prospective, real-world cohort of adults initiating GLP-1 RA OMM therapy, treatment over 24 weeks was associated with significant reductions in body weight, adiposity, and estimated SMM, without significant improvement in overall diet quality or food cravings at the group level. Preservation of muscle-related outcomes during GLP-1 RA OMM therapy remains an important clinical consideration during weight loss [27]. Higher absolute protein intake was modestly associated with preservation of estimated SMM, suggesting that nutrition-focused strategies may play an important role during pharmacologically induced weight loss [13]. However, this association should be interpreted cautiously given the small sample size and borderline statistical significance.

Protein intake normalized to body weight averaged 0.90 ± 0.31 g/kg/day. Although this exceeded the recommended dietary allowance for protein (0.8 g/kg/day), which is intended to meet the minimum protein requirements of healthy adults [28], it remained below the higher protein intakes (1.2–1.6 g/kg/day) increasingly recommended to support lean tissue preservation during intentional weight loss [13,[29], [30], [31]]. Accordingly, the protein intake observed in our cohort was below these proposed targets and is consistent with recent reports that individuals receiving GLP-1 RA therapies may not consume sufficient protein to support preservation of lean muscle mass during weight loss [13,32]. Although some reduction in lean tissue is physiologically expected during weight loss, emerging body composition frameworks emphasize that preservation of muscle-related outcomes during obesity treatment remains clinically important, particularly during rapid pharmacologically induced weight reduction [27,[33], [34], [35]].

Despite substantial weight loss, diet quality did not significantly improve and showed a modest decline over the study period, particularly among participants receiving tirzepatide (Zepbound). While the study was not powered to detect small changes in diet quality, these findings are notable given the established role of dietary quality in cardiometabolic risk reduction and contrasts with the magnitude of physiologic changes observed [[36], [37], [38]]. Together, these results may suggest that reductions in energy intake associated with GLP-1 RA OMM therapy may not necessarily translate to improvements in dietary composition, reinforcing the concept that weight loss and diet quality represent distinct, and not inherently coupled, domains of metabolic health.

The magnitude of weight and fat mass reduction observed in this study is consistent with prior clinical trials of GLP-1 RA OMMs [39]; however, these changes were accompanied by parallel reductions in estimated SMM, with approximately one-quarter of total tissue loss attributable to estimated SMM in our subsample. While some degree of skeletal muscle mass loss is expected during weight reduction [[39], [40], [41], [42]], contemporary body composition frameworks emphasize that reductions in lean mass should not be interpreted as synonymous with direct loss of anatomical SMM in the absence of imaging techniques [27]. Nevertheless, the proportion of estimated SMM reduction observed here raises important clinical considerations, particularly in the absence of targeted dietary or behavioral interventions [35]. These findings align with emerging evidence suggesting that pharmacologically induced weight loss does not selectively preserve lean tissue and may necessitate adjunctive strategies to mitigate potential muscle loss [43,44]. While participants receiving both semaglutide and tirzepatide experienced significant reductions in body weight, fat mass, and estimated SMM, no significant between medication-group differences were observed. These findings should be interpreted cautiously given the exploratory nature of the analysis and the small number of participants receiving semaglutide with body composition data.

The relatively low protein intake observed in our cohort may have contributed to the reductions in BIA-derived estimated SMM seen during GLP-1 RA OMM therapy and supports the ongoing recommendation of nutrition-focused interventions aimed at optimizing protein adequacy during treatment [13].

Exploratory analyses of individual food craving items suggested substantial heterogeneity in craving responses during GLP-1 RA OMM therapy, with no consistent directional pattern across specific food categories. While reductions in select highly palatable foods, such as brownies, were observed, these findings should be interpreted cautiously given the exploratory nature of the analyses. Collectively, these findings may suggest that changes in food cravings during GLP-1 RA OMM therapy are individualized rather than uniformly affecting specific food groups, highlighting the complexity of behavioral responses to pharmacologic weight loss treatment. A recent review of studies to evaluate effects of GLP-1RA OMM therapy on eating behaviors noted that in the limited literature, most report improvement in food cravings and other symptoms, while some remain unchanged [45]. Contrary to recent controlled trial data demonstrating reductions in food cravings and preferences for highly palatable foods during tirzepatide treatment, overall food cravings in our small, real-world cohort did not significantly improve [46]. However, given the limited sample size and variability in craving responses, the absences of a statistically significant change should not be interpreted as evidence of no effect. Additionally, differences in study design, sample size, duration, dietary intervention, and the absence of a controlled feeding condition in our real-world observational study may further explain these discrepancies.

The low prevalence of food insecurity in this cohort limited the ability to evaluate its relationship with dietary intake and suggests that structural barriers to food access were unlikely to be a primary driver of dietary patterns and diet quality was not related to measures of neighborhood food access. Together, these findings suggest that the lack of improvement in dietary quality observed during GLP-1 RA OMM treatment is unlikely to be explained by food access constraints and instead may reflect behavioral factors or pharmacologic effects of therapy. Importantly, participants did not receive structured RDN-led nutrition intervention, reflecting real-world care conditions but potentially contributing to the lack of improvement in diet quality observed.

The observed reduction in total energy intake is consistent with the appetite-suppressive effects of GLP-1 RA OMM therapy and was accompanied by parallel decreases in carbohydrate and fat intake, indicating a global reduction in dietary consumption rather than selective macronutrient shifts. Although the decline in protein intake did not reach statistical significance, the directionality is clinically meaningful, particularly given that absolute protein intake was associated with preservation of estimated SMM, whereas protein intake normalized to body weight was not. These findings suggest that reductions in overall energy intake during GLP-1 RA OMM therapy may inadvertently compromise protein adequacy; however, the observed association between protein intake and estimated SMM should be interpreted cautiously given the potential influence of body size, physical activity, and BIA-estimated SMM measurement.

Similar to recent reports of inadequate micronutrient intake among individuals using GLP-1RA OMM therapies [32,47], participants in our cohort had significant reductions across multiple micronutrients, including magnesium, potassium, iron, zinc, selenium, and several B vitamins, further indicate that decreased energy intake was accompanied by reduced micronutrient exposure. This pattern raises concern for potential intake inadequacy during pharmacologically induced weight loss, particularly in real-world settings where structured nutrition support is not routinely integrated. As participants in this study did not receive RDN intervention, these findings are likely to reflect typical clinical practice and underscore a critical gap in obesity management [35]. Collectively, these results support the need for concurrent nutrition-focused care during GLP-1 RA OMM therapy to preserve diet quality and mitigate unintended nutritional consequences.

## 5 Limitations

These findings should be interpreted within the context of several limitations. The sample size was modest, and recruitment was constrained by real-world factors, including medication access, insurance coverage changes, and cost, limiting statistical power. The study population was predominantly female, which may further limit the generalizability of the findings to male populations. Given the small number of male participants, exploratory sex-stratified analyses were not feasible, and the study was not powered to evaluate potential sex-specific differences in dietary, behavioral, or body composition responses to GLP-1 RA OMM therapy. Attrition was substantial, with approximately one-third of enrolled participants not completing the 24-week assessment. Participants who completed the study had significantly lower baseline BMI than non-completers, raising the possibility of attrition bias and potentially limiting the generalizability of the findings to individuals with higher BMI. If participants with greater obesity experienced different treatment responses, tolerability, or barriers to continued participation, the observed estimates may not fully reflect outcomes in the broader population of individuals initiating GLP-1 RA OMM therapy.

Dietary data were available for a subset of participants and were not missing at random, introducing potential selection bias. Participants with dietary data had lower baseline BMI than those without dietary data, suggesting that the dietary analyses may not fully represent the broader study population. Consequently, estimates of diet quality, nutrient intake, and their associations with body composition outcomes should be interpreted cautiously, as missing dietary data may have influenced both the magnitude and direction of observed findings. Additionally, gastrointestinal side effects associated with GLP-1 RA OMM therapy were not systematically captured, limiting interpretation of whether reductions in energy intake and diet quality were attributable to physiologic appetite suppression, treatment-related symptoms, or both.

Physical activity was assessed in a self-reported questionnaire in a limited subset, restricting interpretation of its role in body composition changes. Body composition was assessed using BIA, which provides indirect estimates of body composition compartments rather than direct imaging-based measures of SMM. Therefore, reductions in estimated SMM should be interpreted cautiously, particularly given that some loss of lean tissue is physiologically expected during weight reduction [27]. The RMR was also estimated using the BIA device and was not directly measured through indirect calorimetry. Consequently, changes in BIA-estimated RMR may be subject to measurement error and should be interpreted with caution [48], as the estimated values may not fully reflect true changes in energy expenditure during treatment. Additionally, as an observational study conducted in a clinical setting, treatment exposure and behavioral factors were not standardized. Given the number of statistical comparisons performed across body composition, dietary, behavioral, and correlation analyses, the possibility of Type I error should be considered. No formal adjustment for multiple comparisons was applied because this study was exploratory and intended to generate hypotheses regarding nutritional and behavioral changes during GLP-1 RA OMM therapy. Accordingly, findings from secondary and exploratory analyses, particularly those with P-values near 0.05, should be interpreted cautiously and require confirmation in larger studies.

Despite these limitations, this study provides novel insight into the behavioral and physiologic adaptations accompanying GLP-1 RA OMM therapy in a real-world setting. A key strength is the use of repeated, real-time dietary assessment across multiple time points using 3-day food records, allowing for characterization of longitudinal dietary patterns during active pharmacologic weight loss. This approach extends beyond prior studies, which have largely relied on cross-sectional intake measures, single time-point assessments, or controlled ad libitum feeding paradigms. The integration of dietary intake, food cravings, and detailed body composition measures addresses a critical gap in the literature, where nutritional outcomes are rarely captured alongside pharmacologic weight loss [7].

## 6 Conclusion

In this prospective real-world study of adults with obesity undergoing 24 weeks of GLP-1 RA OMM therapy, significant reductions were observed in body weight, adiposity, estimated SMM, BIA-estimated RMR, and energy intake. However, diet quality and overall food cravings did not significantly improve despite substantial weight loss. These findings provide novel insight into the nutritional and physiologic adaptations accompanying GLP-1 RA OMM therapy in the absence of structured nutrition intervention.

• Participants lost a mean 9.4% of total body weight over 24 weeks, and 82.6% achieved ≥5% weight loss.
• Weight loss was accompanied by reductions in estimated SMM (−5.0 lb), energy intake (−255 kcal/day), and intake of multiple micronutrients, including magnesium, potassium, iron, and zinc.
• Despite substantial weight loss, HEI-2020 diet quality scores and overall food craving scores did not significantly improve during GLP-1 RA OMM therapy in the absence of structured nutrition intervention.

## Author contributions

D.B. conceived and led the study, including study design, data acquisition oversight, data analysis, and primary drafting of the manuscript. S.T. contributed to participant recruitment and study coordination, including scheduling and administrative logistics. A.K.F. provided clinical and medical oversight. F.M.S. provided senior supervision, guidance on study design, funding acquisition, and critically edited the manuscript. All authors contributed to data interpretation, critically reviewed the manuscript, and approved the final version for submission.

## Ethical adherence and ethical review

This study was a prospective clinical trial involving human participants. All study procedures were conducted in accordance with the ethical standards of the institutional research committee, and the study protocol was approved by the University of California, Davis Institutional Review Board (IRB ID: 2184712-4), and all participants provided written informed consent prior to enrollment. The trial was registered at ClinicalTrials.gov (Identifier: NCT06467604).

## Declaration of artificial intelligence (AI)

Artificial intelligence was not involved in the development, analysis, or writing of the manuscript.

## Funding

USDA CA-D-NTR-6316-H (FS); UC Davis Corrine L. Rustici Endowment in Human Nutrition (FS); UC Davis College of Agriculture and Environmental Science Henry A. Jastro graduate research award (DB); Academy of Nutrition and Dietetics Foundation 2025 Commission on Dietetic Registration Doctoral Scholarship (DB).

## Conflict of interest

A.K.F. reports work on advisory boards for Currax Pharmaceuticals LLC, Eli Lilly, Ms.Medicine, Novo Nordisk, Rhythm Pharmaceuticals, SideKick Health, Seca, and VIVUS.
