# Supplementary material for: Changes in food cravings, dietary quality, body composition, and dietary intake during GLP-1 receptor agonist therapy: The CRAVE study
Source: Obes Pillars. 2026 Jun 29;19:100292. doi: 10.1016/j.obpill.2026.100292 (PMC13334816; doi:10.1016/j.obpill.2026.100292)
Supplement: Multimedia component 1 [file mmc1.docx]

| **Supplemental Table 1. Baseline characteristics of CRAVE participants by availability of dietary intake data used to derive Healthy Eating Index (HEI-2020) scores.** | | | |
| --- | --- | --- | --- |
| **Characteristic** | **HEI Available**  *N=32* | **HEI Not Available**  *N=11* | **P value** |
| **Age, years** | 43.7 (11.9) | 41.1 (11.9) | 0.5 |
| **Sex** |  |  | 0.6 |
| Female | 29 (90.6%) | 9 (81.8%) |  |
| Male | 3 (9.4%) | 2 (18.2%) |  |
| **OMM type** |  |  | 0.2 |
| Semaglutide | 11 (34.4%) | 7 (63.6%) |  |
| Tirzepatide | 21 (65.6%) | 4 (36.4%) |  |
| **Weight, lb** | 204.7 (45.1) | 226.8 (32.3) | 0.051 |
| Missing | 2 | 1 |  |
| **BMI, kg/m²** | 33.7 (6.9) | 37.4 (4.1) | 0.034 |
| Missing | 2 | 1 |  |
| **Waist circumference, in** | 40.8 (5.4) | 43.1 (3.5) | 0.11 |
| Missing | 2 | 1 |  |
| **Fat mass, %** | 44.4 (7.7) | 48.1 (7.2) | 0.2 |
| Missing | 2 | 1 |  |
| **Estimated SMM, lb** | 52.5 (7.4) | 54.4 (14.9) | 0.6 |
| Missing | 2 | 1 |  |
| **BIA- RMR, kcal/day** | 1,647.5 (234.0) | 1,767.7 (248.4) | 0.095 |
| Missing | 2 | 1 |  |
| **Hunger (VAS)** | 35.0 (21.1) | 38.7 (25.1) | 0.6 |
| **Fullness (VAS)** | 49.8 (28.6) | 57.1 (22.6) | 0.5 |
| **Satisfaction (VAS)** | 54.0 (23.3) | 55.6 (17.7) | 0.8 |
| **Desire to eat (VAS)** | 51.2 (24.9) | 50.7 (25.1) | >0.9 |
| Data are presented as mean (SD) for continuous variables and n (%) for categorical variables. Sample sizes vary by variable due to missing data. Dietary intake data were available for a subset of participants due to study design and resource constraints, with processing prioritized for participants who completed at least two study timepoints. Between-group comparisons were performed using Wilcoxon rank-sum tests for continuous variables and Fisher’s exact tests for categorical variables. BMI, Body mass index; OMM, Obesity management medication; RMR, resting metabolic rate; SMM, skeletal muscle mass; VAS, visual analogue scale | | | |

| **Supplemental Table 2. Baseline characteristics of CRAVE participants by 24-week endpoint completion status.** | | | |
| --- | --- | --- | --- |
| **Characteristic** | **Completed endpoint**  *(N = 28)* | **Did not complete endpoint**  *(N = 15)* | **P value** |
| **Age, years** | 44.2 (11.2) | 40.9 (13.1) | 0.5 |
| **Sex** |  |  | >0.9 |
| Female | 25 (89.3%) | 13 (86.7%) |  |
| Male | 3 (10.7%) | 2 (13.3%) |  |
| **OMM type** |  |  | 0.11 |
| Semaglutide (Wegovy) | 9 (32.1%) | 9 (60.0%) |  |
| Tirzepatide (Zepbound) | 19 (67.9%) | 6 (40.0%) |  |
| **Weight, lb** | 204.4 (44.5) | 221.0 (39.2) | 0.12 |
| Missing | 2 | 1 |  |
| **BMI, kg/m²** | 33.6 (7.2) | 36.6 (4.4) | 0.038 |
| Missing | 2 | 1 |  |
| **Waist circumference, in** | 40.7 (5.5) | 42.6 (4.1) | 0.14 |
| Missing | 2 | 1 |  |
| **Fat mass, %** | 44.3 (8.1) | 47.2 (6.8) | 0.3 |
| Missing | 2 | 1 |  |
| **Fat mass, lb** | 93.4 (37.3) | 104.4 (23.4) | 0.12 |
| Missing | 2 | 1 |  |
| **Estimated SMM, lb** | 52.4 (6.5) | 53.9 (13.9) | 0.5 |
| Missing | 2 | 1 |  |
| **BIA- RMR, kcal/day** | 1,645.0 (228.6) | 1,738.1 (258.2) | 0.2 |
| Missing | 2 | 1 |  |
| **HEI-2020 score** | 59.3 (9.8) | 58.7 (15.7) | >0.9 |
| Missing | 0 | 11 |  |
| **Hunger (VAS)** | 34.6 (22.5) | 38.6 (21.3) | 0.6 |
| **Fullness (VAS)** | 48.0 (29.0) | 58.5 (22.5) | 0.2 |
| **Satisfaction (VAS)** | 53.3 (23.5) | 56.5 (18.8) | 0.7 |
| **Desire to eat (VAS)** | 49.6 (26.1) | 53.7 (22.2) | 0.7 |
| Data are presented as mean (SD) for continuous variables and n (%) for categorical variables. Endpoint completion was defined as having data available at the 24-week (final) study timepoint. Sample sizes vary by variable due to missing data. Between-group comparisons were performed using Wilcoxon rank-sum tests for continuous variables and Fisher’s exact tests for categorical variables. BMI, Body mass index; HEI-2020, Healthy Eating Index 2020; OMM, Obesity management medication; RMR, resting metabolic rate; SMM, skeletal muscle mass; VAS, visual analogue scale | | | |

| **Supplemental Table 3.**  Changes in individual Food Craving Inventory (FCI-III) item scores from baseline to 24 weeks during GLP-1 RA OMM therapy (N = 28) | | | | |
| --- | --- | --- | --- | --- |
| **FCI-III Item** | **Baseline** | **Final** | **Change** | **P value** |
| Bacon | 2.286 ± 1.436 | 1.964 ± 1.071 | -0.321 ± 1.389 | 0.237 |
| Biscuits | 1.607 ± 1.031 | 1.321 ± 0.612 | -0.286 ± 0.713 | 0.052 |
| Baked Potato | 1.929 ± 1.184 | 1.714 ± 1.15 | -0.214 ± 1.287 | 0.354 |
| Brownies | 2.321 ± 1.442 | 1.607 ± 0.956 | -0.714 ± 1.675 | 0.043* |
| Cake | 1.857 ± 1.239 | 1.464 ± 0.838 | -0.393 ± 1.397 | 0.141 |
| Canned Fruit | 1.107 ± 0.315 | 1.179 ± 0.548 | 0.071 ± 0.604 | 0.577 |
| Candy | 1.643 ± 0.989 | 1.571 ± 0.92 | -0.071 ± 1.184 | 0.837 |
| Cereal | 1.571 ± 1.103 | 1.393 ± 0.685 | -0.179 ± 0.983 | 0.374 |
| Chips | 1.893 ± 1.315 | 1.893 ± 1.031 | 0 ± 1.054 | 1 |
| Chocolate | 2.214 ± 1.397 | 2.036 ± 1.138 | -0.179 ± 1.492 | 0.491 |
| Cinnamon Rolls | 2.036 ± 1.347 | 1.786 ± 0.957 | -0.25 ± 1.236 | 0.413 |
| Cooked Vegetables | 1.857 ± 1.079 | 2.143 ± 1.177 | 0.286 ± 1.536 | 0.304 |
| Cookies | 2.464 ± 1.427 | 1.821 ± 0.983 | -0.643 ± 1.66 | 0.072 |
| Cornbread | 1.429 ± 0.79 | 1.5 ± 0.745 | 0.071 ± 0.716 | 0.608 |
| Donuts | 1.786 ± 1.287 | 1.5 ± 0.882 | -0.286 ± 1.301 | 0.319 |
| French Fries | 1.857 ± 1.208 | 1.821 ± 1.056 | -0.036 ± 0.999 | 0.968 |
| Fried Chicken | 1.5 ± 0.839 | 1.464 ± 0.838 | -0.036 ± 1.201 | 0.928 |
| Fried Fish | 1.5 ± 1.106 | 1.25 ± 0.585 | -0.25 ± 1.143 | 0.32 |
| Fruit Juice | 1.321 ± 0.67 | 1.536 ± 0.922 | 0.214 ± 0.787 | 0.188 |
| Gravy | 1.179 ± 0.476 | 1.143 ± 0.591 | -0.036 ± 0.693 | 0.572 |
| Hamburger | 1.75 ± 1.11 | 1.786 ± 0.957 | 0.036 ± 1.29 | 0.748 |
| Hot Dog | 1.286 ± 0.659 | 1.143 ± 0.448 | -0.143 ± 0.756 | 0.386 |
| Ice Cream | 2.357 ± 1.162 | 2.143 ± 1.297 | -0.214 ± 1.371 | 0.414 |
| Pancakes/Waffles | 1.714 ± 1.15 | 1.393 ± 0.685 | -0.321 ± 1.124 | 0.174 |
| Pasta | 2.107 ± 1.257 | 1.929 ± 1.12 | -0.179 ± 1.056 | 0.412 |
| Pizza | 2.107 ± 1.397 | 1.821 ± 0.945 | -0.286 ± 1.487 | 0.366 |
| Raw Fruit | 1.857 ± 1.177 | 2.25 ± 1.11 | 0.393 ± 1.166 | 0.068 |
| Raw Vegetables | 1.643 ± 0.951 | 2 ± 1.089 | 0.357 ± 0.989 | 0.098 |
| Rice | 1.607 ± 1.031 | 1.964 ± 1.232 | 0.357 ± 1.162 | 0.119 |
| Rolls | 1.714 ± 1.117 | 1.5 ± 0.923 | -0.214 ± 1.031 | 0.275 |
| Sandwich Bread | 1.714 ± 0.937 | 1.75 ± 1.041 | 0.036 ± 1.138 | 0.902 |
| Sausage | 1.5 ± 0.793 | 1.357 ± 0.621 | -0.143 ± 0.97 | 0.385 |
| Steak | 1.857 ± 1.38 | 2.036 ± 0.999 | 0.179 ± 1.416 | 0.491 |
| Values are mean ± SD. Change was calculated as final minus baseline; negative values indicate reduced cravings. P values were calculated using paired Wilcoxon signed-rank tests. Item-level analyses were exploratory and not adjusted for multiple comparisons. | | | | |

| **Supplemental Table 4.** Changes in dietary intake and nutrient composition from baseline to 24 weeks during GLP-1 RA OMM therapy (N=28) | | | | |
| --- | --- | --- | --- | --- |
|  | **Baseline** | **Endpoint** | **Change** | **P value** |
| **Energy and Macronutrients** |  | | | |
| Energy (kcal) | 1565.79 ± 473.77 | 1310.54 ± 316.67 | -255.26 ± 490.17 | 0.008* |
| Total protein (g) | 86.2 ± 23.17 | 77.47 ± 21.02 | -8.73 ± 26.44 | 0.094 |
| Animal protein (g) | 62.2 ± 18.84 | 56.87 ± 17.29 | -5.33 ± 23.28 | 0.269 |
| Plant protein (g) | 24 ± 10.95 | 20.6 ± 7.61 | -3.41 ± 9.66 | 0.06 |
| Total fat (g) | 66.85 ± 25.04 | 55.59 ± 16.97 | -11.26 ± 25.55 | 0.037* |
| Saturated fat (g) | 20.5 ± 8.11 | 17.88 ± 6.01 | -2.62 ± 9.05 | 0.19 |
| Monounsaturated fat (g) | 24.15 ± 11.81 | 19.07 ± 6.71 | -5.08 ± 11.76 | 0.021* |
| Polyunsaturated fat (g) | 15.45 ± 6.27 | 13.01 ± 5.56 | -2.44 ± 7.25 | 0.057 |
| Total carbohydrate (g) | 155.23 ± 55.38 | 130.63 ± 39.94 | -24.61 ± 58.72 | 0.014* |
| Carbohydrate (% energy) | 37.67 ± 6.26 | 37.82 ± 6.24 | 0.16 ± 8.29 | 0.847 |
| Protein (% energy) | 23.40 ± 5.87 | 24.68 ± 5.15 | 1.28 ± 6.60 | 0.531 |
| Fat (% energy) | 37.19 ± 5.38 | 37.04 ± 5.72 | -0.15 ± 7.14 | 0.991 |
| **Carbohydrate Quality** |  | | | |
| Total sugars (g) | 53.22 ± 25.18 | 45.43 ± 22.29 | -7.79 ± 22.75 | 0.031* |
| Added sugars (g) | 25.45 ± 16.66 | 21.12 ± 14.11 | -4.34 ± 17.29 | 0.108 |
| Added sugars (% total sugars) | 23.92 ± 15.99 | 19.73 ± 13.61 | -4.19 ± 16.29 | 0.094 |
| Total dietary fiber (g) | 19.88 ± 8.94 | 17.2 ± 7 | -2.68 ± 7.75 | 0.086 |
| Starch (g) | 69.69 ± 28.96 | 57.97 ± 23.82 | -11.72 ± 36.71 | 0.082 |
| **Fatty Acid Profile** |  | | | |
| Total trans fat (g) | 0.88 ± 0.38 | 0.81 ± 0.34 | -0.07 ± 0.49 | 0.562 |
| Omega-3 fatty acids (g) | 1.77 ± 0.81 | 1.65 ± 1.07 | -0.12 ± 0.99 | 0.29 |
| Omega-6 fatty acids (g) | 13.13 ± 5.43 | 10.86 ± 4.62 | -2.26 ± 6.4 | 0.067 |
| **Micronutrients** |  | | | |
| Vitamin D (mcg) | 3.82 ± 3.73 | 4.71 ± 5.17 | 0.89 ± 6.54 | 0.657 |
| Vitamin B12 (mcg) | 3.3 ± 1.39 | 2.98 ± 1.12 | -0.31 ± 1.79 | 0.406 |
| Folate (DFE, mcg) | 375.21 ± 126.98 | 325.2 ± 109.7 | -50.01 ± 123.89 | 0.035* |
| Calcium (mg) | 847.91 ± 416.06 | 757.39 ± 347.24 | -90.52 ± 372.6 | 0.311 |
| Magnesium (mg) | 283.77 ± 108.04 | 236.42 ± 85.8 | -47.35 ± 83.19 | 0.005* |
| Iron (mg) | 11.24 ± 5.38 | 9.33 ± 2.62 | -1.92 ± 5.28 | 0.044* |
| Heme iron (mg) | 0.67 ± 0.46 | 0.62 ± 0.31 | -0.06 ± 0.54 | 0.811 |
| Non-heme iron (mg) | 10.57 ± 5.38 | 8.71 ± 2.66 | -1.86 ± 5.24 | 0.046* |
| Potassium (mg) | 2326.31 ± 707.62 | 1945.82 ± 691.74 | -380.49 ± 687.85 | 0.007* |
| Sodium (mg) | 2918.06 ± 770.87 | 2425.03 ± 671.79 | -493.03 ± 962.29 | 0.014* |
| Zinc (mg) | 8.85 ± 2.8 | 7.62 ± 1.88 | -1.23 ± 2.99 | 0.049* |
| Copper (mg) | 1.2 ± 0.45 | 0.98 ± 0.31 | -0.22 ± 0.35 | 0.003* |
| Selenium (mcg) | 94.23 ± 25.51 | 81.32 ± 25.44 | -12.91 ± 31.78 | 0.046* |
| Manganese (mg) | 2.83 ± 1.39 | 2.3 ± 0.93 | -0.53 ± 1.28 | 0.020* |
| Vitamin A (RAE, mcg) | 1024.09 ± 620.2 | 792.9 ± 454.14 | -231.19 ± 642.6 | 0.025* |
| Vitamin C (mg) | 65.86 ± 37.82 | 69.55 ± 50.75 | 3.69 ± 52.16 | 0.811 |
| Vitamin K (mcg) | 214.71 ± 192.38 | 129.35 ± 105.23 | -85.36 ± 206.32 | 0.028* |
| Thiamin (mg) | 1.25 ± 0.47 | 1.14 ± 0.39 | -0.12 ± 0.47 | 0.241 |
| Riboflavin (mg) | 1.48 ± 0.49 | 1.33 ± 0.5 | -0.15 ± 0.51 | 0.052 |
| Niacin (mg) | 21.4 ± 6.07 | 17.42 ± 4.96 | -3.98 ± 6.52 | 0.006* |
| Niacin equivalents (mg) | 40.16 ± 9.91 | 34.58 ± 8.86 | -5.58 ± 10.6 | 0.013* |
| Pantothenic acid (mg) | 4.75 ± 1.33 | 3.85 ± 1.23 | -0.9 ± 1.36 | 0.002* |
| Vitamin B6 (mg) | 1.86 ± 0.84 | 1.43 ± 0.52 | -0.43 ± 0.8 | 0.002* |
| **Grains** |  | | | |
| Total grains (oz eq) | 4.51 ± 2.26 | 3.75 ± 1.97 | -0.76 ± 2.6 | 0.136 |
| Whole grains (oz eq) | 1.06 ± 1.12 | 0.6 ± 0.62 | -0.46 ± 1.31 | 0.26 |
| Refined grains (oz eq) | 3.45 ± 1.79 | 3.14 ± 1.8 | -0.31 ± 1.92 | 0.516 |
| **Other Dietary Components** |  | | | |
| Caffeine (mg) | 73.98 ± 60.69 | 62.1 ± 51.91 | -11.88 ± 39.99 | 0.119 |
| Values are presented as mean ± SD. Changes represent within-participant differences from baseline to 24 weeks. P values were calculated using paired statistical tests. Nutrient intakes were derived from 3-day food records collected at each time point and analyzed using Nutrition Data System for Research. Diet quality proxies (grain intake) are expressed as ounce equivalents (oz eq). DFE, dietary folate equivalents; RAE, retinol activity equivalents. * P < 0.05 | | | | |

| **Supplemental Table 5.** Changes in anthropometric and body composition measures from baseline to 24 weeks during GLP-1 RA OMM therapy (N = 23) | | | | |
| --- | --- | --- | --- | --- |
|  | **Baseline** | **Final** | **Change** | **P value** |
| Weight, lb | 198.5 (35.4) | 179.5 (32.3) | -19 (11.9) | <0.001 |
| Total body weight loss, % |  |  | 9.4 (5.0) |  |
| BMI, kg/m^2^ | 32.7 (6.1) | 29.6 (5.7) | -3.1 (2.0) | <0.001 |
| Waist circumference, in | 40.4 (5.5) | 36.1 (5.5) | -4.3 (2.3) | <0.001 |
| Fat mass, % | 43.6 (7.9) | 40.3 (8.6) | -3.4 (2.6) | <0.001 |
| Fat mass, lb | 88.8 (30.6) | 74.4 (28.8) | -14.4 (10.1) | <0.001 |
| Estimated SMM, lb | 52.0 (6.3) | 47.0 (7.0) | -5.0 (5.3) | <0.001 |
| BIA-estimated RMR, kcal/day | 1611.9 (189.6) | 1523.7 (172.8) | -88.2 (51.8) | <0.001 |
| Values are presented as mean (SD). Change represents within-subject difference from baseline to endpoint. P values were calculated using paired t-tests. BIA, body impedance analysis; BMI, body mass index; RMR, resting metabolic rate; SMM, skeletal muscle mass. | | | | |

| **Supplemental Table 6.** Changes in body weight and body composition measures from baseline to 24 weeks by GLP-1 RA OMM | | | |
| --- | --- | --- | --- |
| Outcome | Wegovy (n=6) | Zepbound (n=17) | Between group P-values |
| Weight change, lb | -15.5 (-25.9, -5.2) | -20.2 (-26.7, -13.8) | 0.37 |
| Estimated SMM, lb | -4.2 (-7, -1.4) | -5.3 (-8.4, -2.3) | 0.534 |
| Fat mass change, lb | -12 (-22, -2) | -15.2 (-20.6, -9.9) | 0.501 |
| Values represent mean change from baseline to 24 weeks with 95% confidence intervals (CI) shown in parentheses. Negative values indicate reductions from baseline. Between-group P values compare change scores between participants receiving semaglutide (Wegovy) and tirzepatide (Zepbound) using independent-samples t tests. These exploratory subgroup analyses were not powered to detect differences between medication groups. SMM, skeletal muscle mass. | | | |

*
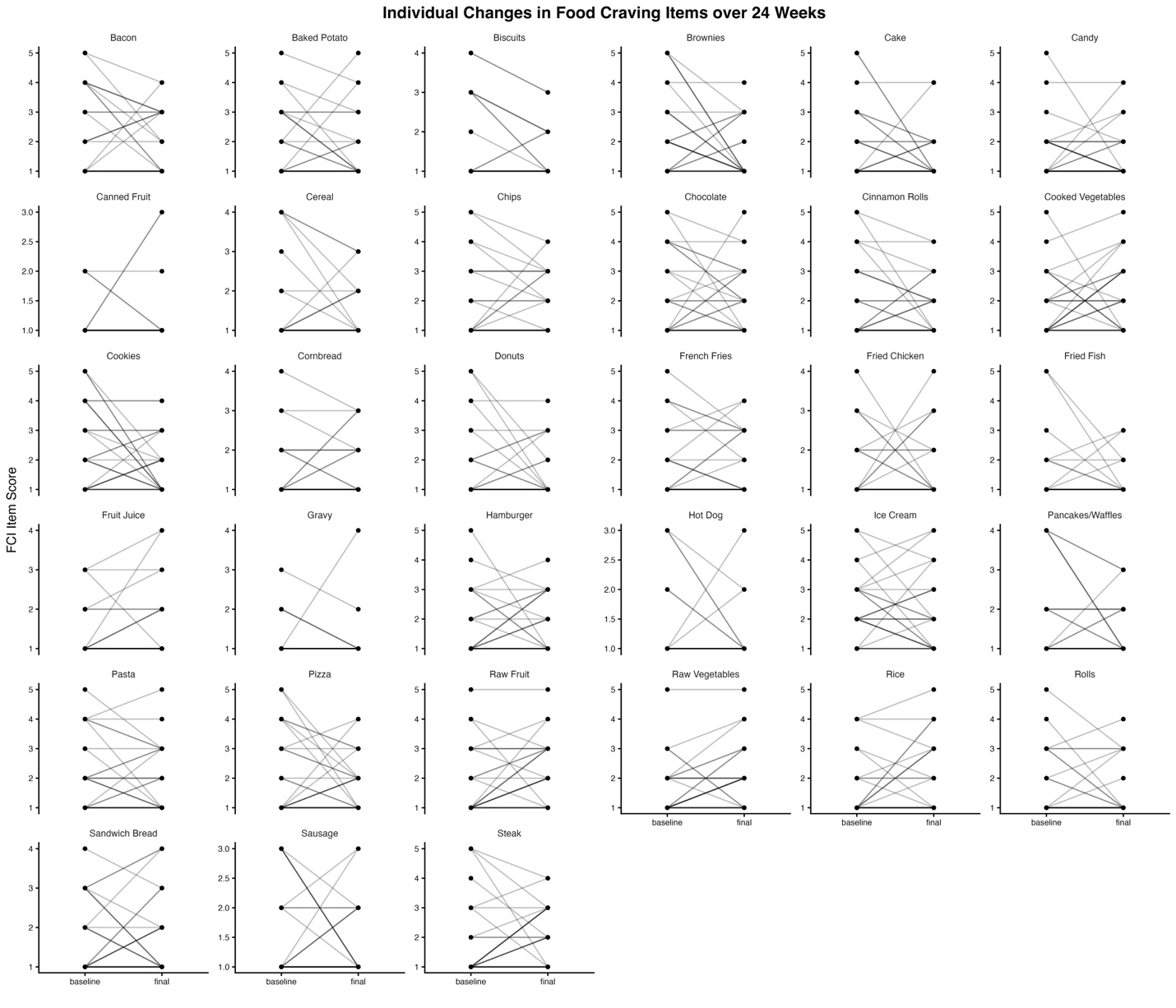
*

Supplemental Figure 1. Each line represents an individual participant with paired baseline and 24-week FCI item scores. Considerable inter-individual variability in craving responses was observed across food categories, without a consistent directional pattern across specific items.


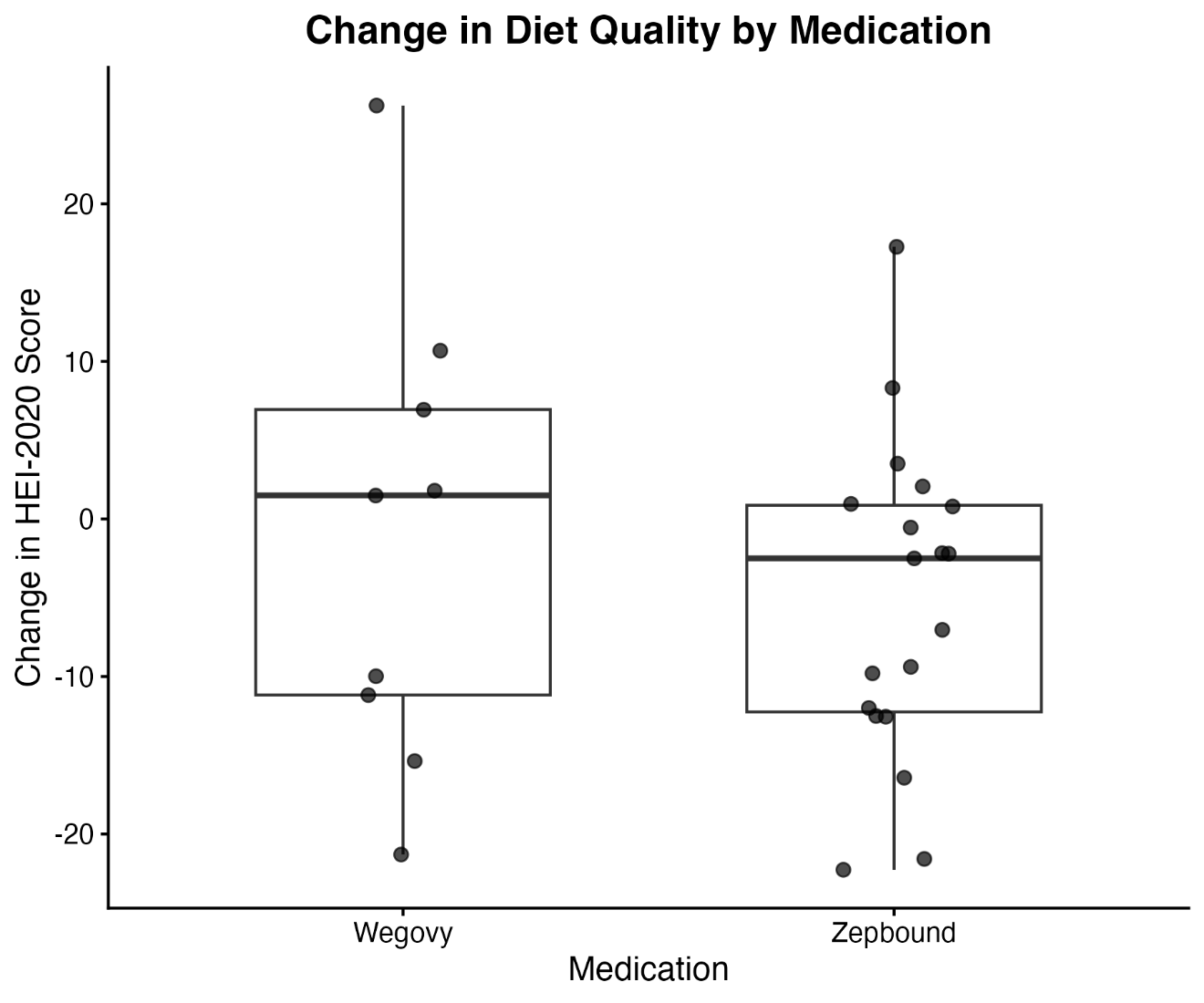


Supplemental Figure 2 Boxplots display median and interquartile range (IQR), with whiskers representing 1.5× IQR. Points represent individual participants. Change in HEI-2020 score reflects within-subject difference from baseline to 24 weeks, with positive values indicating improvements in diet quality. Wegovy (semaglutide); Zepbound (tirzepatide).
